# Pathogens, Social Networks, and the Paradox of Transmission Scaling

**DOI:** 10.1155/2011/267049

**Published:** 2011-03-09

**Authors:** Matthew J. Ferrari, Sarah E. Perkins, Laura W. Pomeroy, Ottar N. Bjørnstad

**Affiliations:** ^1^Center for Infectious Disease Dynamics, Department of Biology, The Pennsylvania State University, University Park, PA 16802, USA; ^2^Cardiff School of Biosciences, Cardiff University, Biomedical Sciences Building, Museum Avenue, Room C7.29, Cardiff CF10 3AX, UK; ^3^Department of Veterinary Preventative Medicine, The Ohio State University, Columbus, OH 43210, USA; ^4^Center for Infectious Disease Dynamics, Departments of Biology and Entomology, The Pennsylvania State University, University Park, PA 16802, USA

## Abstract

Understanding the scaling of transmission is critical to predicting how infectious diseases will affect populations of different sizes and densities. The two classic “mean-field” epidemic models—either assuming density-dependent or frequency-dependent transmission—make predictions that are discordant with patterns seen in either within-population dynamics or across-population comparisons. In this paper, we propose that the source of this inconsistency lies in the greatly simplifying “mean-field” assumption of transmission within a fully-mixed population. Mixing in real populations is more accurately represented by a network of contacts, with interactions and infectious contacts confined to the local social neighborhood. We use network models to show that density-dependent transmission on heterogeneous networks often leads to apparent frequency dependency in the scaling of transmission across populations of different sizes. Network-methodology allows us to reconcile seemingly conflicting patterns of within- and across-population epidemiology.

## 1. Introduction

Transmission is the driver of host-pathogen interactions and the most important determinant of disease dynamics. The patterns and dynamics of transmission within any given host population depend on how infectious and susceptible hosts interact, both spatially and socially [1]. Ultimately, pathogen transmission at the population level is determined by patterns of mixing at the individual level. Network science has highlighted that the mean and variance in transmission among individuals is key to the dynamics of spread within a given population network [2–5]. Predictions of disease dynamics, however, often require that we make projections from one population to another. Population size (i.e., counts), and possibly density, is a common metric with which to characterize differences among populations. Thus, a central challenge in infectious disease ecology is to understand how the distribution of contacts scales across populations of varying size.

Classically, the complex biology of host mixing has been characterized using select formulations from a small number of candidate mathematical models (e.g., [6–11]). If we consider only the increase in infected individuals (i.e., due to disease transmission), then the rate of increase in the number of infected individuals (*I*), *dI*/*dt*, is given by the number of susceptible hosts, *S*, times the force of infection (the per capita rate of infection). The force of infection, in turn, is the product of the rate of contacts among individuals, the probability of a contact being with an infectious host, and the probability of that contact giving rise to an infection. The first and third terms are commonly combined into a transmission term,  *β*. In the simplest case, the rate of contact among hosts is assumed constant (i.e., independent of both population size and density). In a well-mixed population, the probability of any given contact being infected is then *I*/*N*, where *N* is the population size (i.e., count), and the rate of increase of infected individuals in the population is given by

(1) $$\begin{array}{c} \frac{dI}{dt}=\beta S\frac{I}{N}. \end{array}$$

This formulation is commonly referred to as *frequency-dependent transmission* (note that there has been extensive discussion of the appropriate terminology for these models; here, we defer to the derivations and terminology of Begon et al. [10]). Alternatively, if transmission scales linearly with population density (i.e., *β* = *θN*/*A*, where *A* is area occupied by the population) then we arrive at the *density-dependent transmission* model:

(2) $$\begin{array}{c} \frac{dI}{dt}=\beta SI. \end{array}$$

Though the derivation of (2) is stated in terms of population density, the dependence on area is commonly suppressed by assuming that area is constant through time [10], and thus population size and density are equivalent measures. Begon et al. [10] caution, however, that this simplification in interpreting the two models leads to challenges in comparing dynamics across different populations that presumably occupy ranges of a different area. The frequency- and density-dependent transmission models are extreme special cases. While a variety of alternative or intermediate models have been proposed on theoretical or empirical grounds (see [9–11]), these remain the dominant archetypes in the literature.

Both models assume homogeneous mixing (“mean field”) with no explicit spatial or social structure. While the mechanism of transmission is not explicitly considered in the derivation of (1) and (2), the choice between these two models is often motivated by the mode of transmission. Vector-borne and sexually transmitted diseases are generally assumed to be transmitted in a frequency-dependent manner because the mean number of contacts is independent of population density (if the number of sexual partners or vector attack rates are constant, e.g., [12]). Directly transmitted diseases, in contrast, are typically expected to spread in a density-dependent manner (because the number of encounters may increase with density and/or population size). Alternatively, the density-dependent transmission model has been equated with homogeneous or uniformly random mixing among individuals in contrast to frequency-dependent transmission, which is taken to reflect some degree of local heterogeneity in the population (i.e., in sexual partners) [13]. Begon et al. [10], however, have argued that heterogeneity of the contact structure is orthogonal to the distinction between density- and frequency-dependent transmission.

For host populations of constant size, occupying ranges of constant area, and pathogens that do not cause mortality, the frequency- and density-dependent formulations are equivalent. One may think of both as having a force-of-infection (=*per* susceptible rate of infection) equal to *β*′*I*, where *β*′ = *β*/*N* for the former and *β*′ = *β* for the latter. However, they make very different predictions about dynamics and control—such as targets for vaccination coverage and culling [9, 14]—when the host population size varies as a result of extrinsic forces or disease-induced mortality because of their implicit scaling-laws. In the frequency-dependent model, the realized population-level *per capita *(per susceptible-and-infected) transmission rate ($\hat{\beta }=dI/SIdt$) declines with increasing population size (*N*). As a consequence, the basic reproductive ratio, *R*_0_—which defines the epidemic invasion criterion (*R*_0_ > 1)—remains constant across varying population sizes. In contrast, the density-dependent model predicts a constant per capita transmission rate across population density. In this model, $\hat{\beta }$ is independent of population size, so consequently *R*_0_ increases with *N*. The difference in the predictions of the two models leads to important and divergent predictions for dynamics: the frequency-dependent model has no threshold density for invasion [15] and predicts that moderately infectious pathogens (*R*_0_ > 2) that result in lethal infections should lead to extinction of the host and the pathogen [14, 16] while the density-dependent model predicts a critical host density for pathogen invasion and long-term persistence and coexistence of the host and pathogen [17]. Further, for endemic, immunizing pathogens of hosts with a relatively long lifespan, *L*, the mean age-of-infection is predicted to be approximately *L*/*R*_0_ [17, 18]. Thus, based on these models, we expect that frequency-dependent pathogens should exhibit constant mean age-of-infection and proportion of the population that is susceptible while, with density-dependent pathogens, mean age-of-infection and susceptible proportion decay with host population size.

Though the theoretical predictions of the frequency- and density-dependent transmission models are clearly distinct, the empirical patterns that emerge when transmission rates (or *R*_0_) have been estimated in real populations are less clear [11]. When reviewing the literature, we find that the patterns do not align with the classic dichotomy between directly transmitted pathogens and sexually or vector transmitted pathogens (Table 1). The different theoretical predictions with respect to the scaling of dynamics with population size are particularly relevant, as we often observe disease processes at one scale (or location) and make inference about the behavior at another. As such, understanding how transmission scales across populations of different sizes are critical to making valid predictions. 

**Figure 1:**
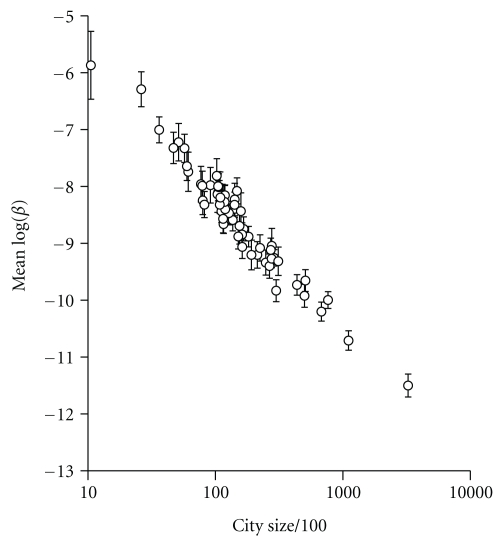
Scaling of measles transmission. The estimated mean transmission rate (*β*) of measles in England and Wales plotted against increasing city size in thousands. Reproduced from [19].

Numerous empirical observations have provided direct measures of *R*_0_ and/or $\hat{\beta }$ from collections of host populations that vary in size geographically, or individual populations that vary in size through time (Table 1). For example, two directly transmitted pathogens within the morbilliviridae have had $\hat{\beta }$ estimated for a broad range of population sizes: both measles [19] (Figure 1) and phocine distemper virus [20] found $\hat{\beta }$ to be inversely related to population size, and as a consequence, *R*_0_ to be relatively invariant. These scaling patterns are as predicted by the frequency-dependent model, despite these pathogens being directly transmitted (and not “frequency-dependent” STDs or vector-borne pathogens) for which density dependence is normally expected. Other empirical studies are ambiguous in their model support when scaled across populations (Table 1). For example, *R*_0_ was concluded to be invariant across population sizes for Aujeszky's disease virus in pigs [21], supporting a frequency-dependent model; Begon et al. [22] found equal support for frequency- and density-dependent transmission models in cowpox data in rodents; Smith et al. [11] have subsequently found support for model that is intermediate to the density- and frequency-dependent models based on an analysis of long-term time series from the same system. Bucheli and Shykoff [23] argued that support of the density- versus frequency-dependent model depended on the spacing of the host plants in pollinator-vectored anther smut. Klepac et al. [24] found that while the density-dependent model fit the observations of the 2002 phocine distemper virus outbreak in the Dutch Wadden Sea better than a frequency-dependent model, the observed dynamics in juvenile and adult seals (which are less social) was better explained by a frequency-dependent model. 

Experimental manipulations of populations and studies of the ensuing scaling patterns are also equivocal in their support for either frequency or density dependence. Knell et al. [25, 26] found that transmission of *Bacillus thuringiensis* and a granulosis virus increased with density of susceptible *Plodia interpunctella* and decreased with density of infectious cadavers, and thus fails to conform to the density-dependent model. Antonovics and Alexander [27] manipulated both host density and frequency of infected *Silene latifolia* and found that deposition of the anther smut fungus *Microbotryum violaceum* by pollinating insects increased with frequency of infection, but not density, supporting the notion that vector-borne pathogens are spread in a frequency-dependent fashion. Ryder et al. [28], however, independently manipulated the density and frequency of two-spot lady birds, *Adalia bipunctata*, parasitized by the mite, *Coccipolipus hippodamiae*, and found that infection rates scaled with host density because of increased promiscuity at high density, in contrast to the notion that STDs spread in a frequency-dependent fashion.

Evidence based on serology and age-serology is also difficult to interpret, yet the serological data for a range of directly transmitted human pathogens [17] is to a greater or lesser extent consistent with an invariant *R*_0_ across scales and thus, in accordance with the frequency-dependent model. Edmunds et al. [29] found the median age at infection for Hepatitis B virus to vary from 1–18 years in highly endemic areas (6 African surveys, 3 in south-east Asia, 2 in Oceania, and 1 in South American). These surveys spanned populations ranging in size from a few hundred (an Amerindian village; [30]) to >10 million (Hu et al. [31]) and showed no negative correlation between median age and population size. Metcalf et al. [32] similarly found no correlation between mean age of infection with rubella and population size in the 31 states of Mexico and Mexico City. 

On the whole, scaling of $\hat{\beta }$ and *R*_0_ across populations tend to follow predictions consistent with the frequency-dependent model. In contrast, dynamical patterns within populations are often contradictory to this model and favor the density-dependent model. A key distinction between the frequency- and density-dependent models is that the former predicts moderately lethal pathogens can lead to extinction of the host and parasite. Rachowicz and Briggs [33] showed that transmission of *Batrachochytrium dendrobatidis*, which has been implicated in amphibian extinctions, in tadpoles more closely scaled with frequency than density of infected individuals. In general, however, there is little empirical evidence of pathogen-induced host extinction. In a review of 43 empirical papers, de Castro and Bolker [14] found only one study that gives direct evidence of pathogen-induced extinction. McCallum et al. [34] observed the maintenance of high prevalence of the directly transmitted devil facial tumor disease even as populations suffered significant declines, raising the concern that the disease could lead to extinction of the host. Under the density-dependent model, the reduction of the susceptible population below a threshold level drives the effective transmission rate below 1 and leads to extinction of the pathogen only. The successful application of this principle in, for example, rabies [35], smallpox [36], and foot-and-mouth disease [37] is consistent with density-dependent transmission. 

Measles and phocine distemper, which have been well studied both within and across populations, are exemplary of the paradoxical predictions of the mixing models; both exhibit scaling of transmission rates across populations that is consistent with frequency dependence but local dynamics that are consistent with density dependence [19]. We believe that this paradox arises from the application of the same mean-field transmission model to describe both the within-population transmission and the implicit scaling between populations. We need to revisit the assumption of random host mixing in the face of the strong social and spatial structuring that may limit the interactions between individuals [1]. The dynamics of within-population transmission depend on both the mode of transmission and the structure of transmission network [2–4]; however, the scaling of transmission across populations hinges on the social and spatial structuring of the host population, somewhat independent of the mode of transmission. Note that this point was already raised by De Jong et al. [15]. In the next section, we use network models to show that the equivocal support of the classical models can be resolved by explicit considerations of the contact networks of social and spatial contact patterns.

## 2. Epidemics and Social Networks

Network models have become very popular methods to relax the assumption of complete mixing among individuals (see [5] for a general review). In most natural populations and particularly those with strong social or spatial structure, individuals interact with only a small proportion of the population. We first introduce the network formalism to capturing social organization, and then we discuss the emergent scaling of pathogen transmission on three classes of social networks. 

Epidemic network models differ from the mean-field models in that individuals only interact within their local social neighborhood. We can use these models to investigate the scaling of the realized *per capita* transmission rate, $\hat{\beta }$, with host population size in relation to both the mean neighborhood size (〈*k*〉 = mean  degree) and the heterogeneity of contacts. Cellular automata models have similarly been used to study the distinction between local transmission and global dynamics (e.g., [38–41]). However, the nature of cellular automata models limit the range of social structures that can be studied to those that can be reasonably collapsed to 2 dimensions (i.e., a lattice). Contact network models allow the flexibility to study a wide range of social structures with a complex mix of both local and global interactions [42]. 

Various characteristics of contact network topology (i.e., clustering, assortativity, etc.) may vary with population size. However, there are few empirical studies of the scaling of network properties on social networks of various sizes. Here, we focus simply on the scaling of the mean number of contacts with network size, both because of its straight forward interpretation and its parallels with the classic formulations of the density- and frequency-dependent mean-field models. The classic mean-field models make explicit assumptions about the way the mean rate of contacts scales with population density, but are restricted by implicit assumptions of how the variance in contacts scales with density. Contact network models make explicit the relationship between the mean and variance of contacts in the choice of the degree distribution. Further, contact network models relax the assumption that population density or size is a proxy for the contact rate, which is implicit in the mean-field models. 

**Figure 2:**
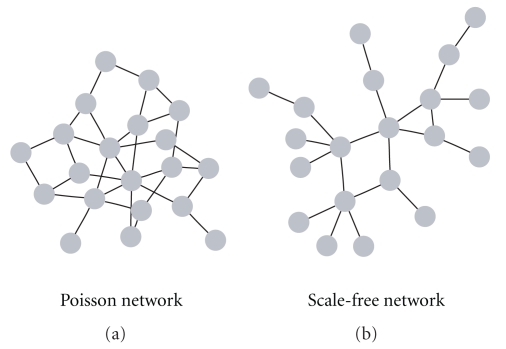
Classes of social networks. Two classes of social networks where the node represents an individual and the edge a social connection or epidemiological relevant contact according to edge distributions (i.e., contact networks) that are described by (a) Poisson networks and (b) power law networks.

Host social organizations that have constraints on group size (e.g., classroom size for school children, harem size for some social mammals) may be adequately represented by a Poisson distribution of social contacts (i.e., a variance to mean ratio near 1, Figure 2(a)) [2]. In contrast, many animal species exhibit skewed social contact networks with few individuals having many contacts, and the majority having few contacts (i.e., a variance to mean ratio ≫ 1, Figure 2(b)) [43, 44]. Following established theory, these social interactions are commonly characterized by truncated power laws [45, 46]. 

We generated networks of size *N* = 100, 500, 1000, 2000, 5000, and 10000 nodes (individuals) for which the mean number of contacts scaled in one of 3 ways: (1) the mean number of contacts was independent of population size (〈*k*〉 = 6), (2) the mean number of contacts increased slower than linearly with population size ($\langle k\rangle =.6*\sqrt{N}$), and (3) the mean number of contacts increased linearly with population size (〈*k*〉 = 0.06∗*N*). Note that all networks have the same mean when *N* = 100. The constant and linear scaling functions are analogous to the assumptions of the classic frequency- and density-dependent transmission functions in the mean-field models when area is held constant. The intermediate function represents an intermediate setting where, for example, the number of contacts increases initially with population size (e.g., larger cities lead to larger workplaces), but the total number of contacts is limited by time or typical home range. In these models, we base the scaling of contacts on the population size rather than density. While in many settings it may be reasonable to assume that the probability of a contact depends on spatial proximity [47], the complexity of empirical contact networks has highlighted that space is not always a reasonable proxy for social proximity [48].

We considered the effect of local heterogeneity of contacts by generating networks with Poisson, exponential, and truncated power law contact distributions. The Poisson contact distribution reflects a setting with relatively low variance in the number of contacts and thus approximates the homogeneous contact structure of the mean-field models [10]. The power law contact distributions reflect extreme heterogeneity in local contacts that is seen in many empirical contact networks. The exponential contact distribution represents an intermediate case. Networks were generated using the algorithm described by Molloy and Reed [49]. We approximated a power law degree distribution using a negative binomial distribution with dispersion parameter, *θ* = 0.1. The presence of highly connected individuals, so-called “super spreaders” [43], has been consistently shown to have large impacts on the threshold conditions and final size of outbreaks [4, 50]. Thus, under the assumption of constant mean contacts for networks of increasing size, we might expect emergent scaling of dynamics for the three degree distributions as larger networks will better sample the degree distribution, which would mean a greater proportion of rare “super spreaders” in the exponential and power law distributed networks.

For all three families of edge distributions, nodes were connected at random with the restriction that self-loops (nodes connected to themselves) and double edges between nodes were disallowed. Thus, individuals cannot infect themselves and cannot infect another in the population more than once. 

We simulated epidemics on the contact networks according to a discrete time, stochastic susceptible-infected-removed (“chain-binomial”) [51] model. Susceptible hosts become infected in each time step with probability $p_{j}=1-\exp\;(-\beta {\overset{̅}{I}}_{j})$, where ${\overset{̅}{I}}_{j}$ is the number of infected individuals within the local neighborhood of individual *j* at a given time point. Thus, infection depends on the local density of infected hosts, where density is relative to the social neighborhood rather than a fixed neighborhood in Euclidean space (i.e., transmission is *locally* density dependent). Infected individuals were removed from the population in each time step with probability 1 − exp (−*γ*), thus leading to a geometrically distributed infectious period—the discrete time equivalent of the standard SIR assumptions. Removed individuals were assumed to be permanently immune and thus unable to be subsequently infected; in practice, these nodes in the network are “turned off”, so while the total number of nodes remains constant, the effective number of nodes in the epidemiologically active portion of the network declines (i.e., thus, for this model equivalent to disease-induced mortality). In an important contrast to the mean-field models, the removal of infected nodes results in reduced contacts for their remaining neighbors. Thus, for structured contact networks (as has been well explored for cellular automata models [38, 39]), the local and global impact of the removal of infected individuals can be quite different. However, in contrast to nonnetwork models (e.g., cellular automata and small-world models for which only local connections are explicit), variance in the distribution of contacts leads to a structured cascade of infection from highly connected individuals to less well-connected individuals, which results in a decline in the per capita realized transmission. This effect is equivalent to the frailty effect discussed in mathematical demography [19]. The rate and magnitude of this decline depends explicitly on the distribution of the underlying transmission network [52]. 

For each configuration, we generated 30 networks and simulated epidemics seeded by a single infection. In each time step, the probability of transmission across an edge was assumed to be 0.1, and the probability that an infected node recovered was 0.1. We calculated, for each configuration and each time step, the realized per capita (per infected) transmission rate, ${\hat{\beta }}_{t}$:

(3) $$\begin{array}{c} {\hat{\beta }}_{t}=\frac{I_{t}-I_{t-1}}{S_{t}I_{t}}, \end{array}$$

where *I*_*t*_ and *S*_*t*_ are the total number of infectious and susceptible nodes on the network at time *t*. The selective removal of highly connected nodes early in the epidemic results in a decrease in the realized transmission rate as the epidemic progresses [52]. To generate time-weighted realized per capita rates, we, therefore, calculated $\hat{\beta }$ as the intercept of the regression of ${\hat{\beta }}_{t}$ on time. Thus, we are describing the scaling of the rate of transmission at the initiation of an epidemic. Calculations using the time-course average yields similar scaling results and are, therefore, not included (Ferrari et al., unpublished results).

**Figure 3:**
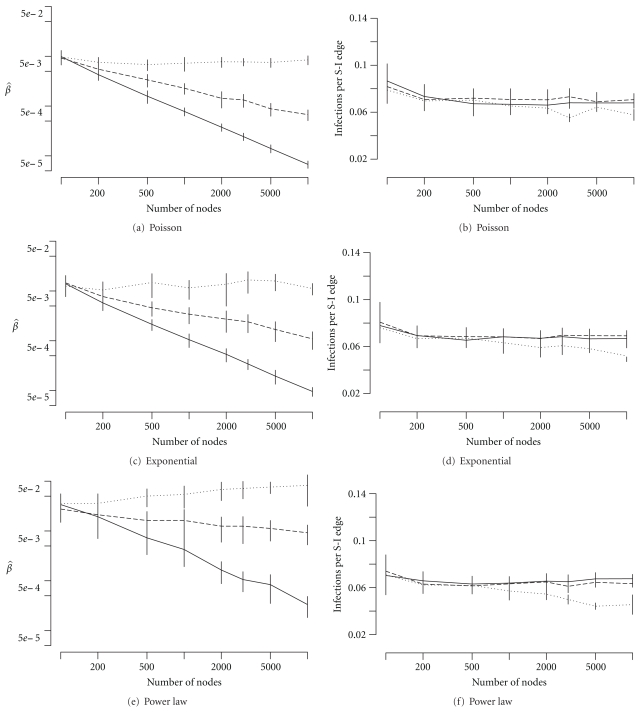
Scaling of transmission on Poisson (a, b), exponential (c, d), and scale-free (e, f) networks. Left-hand panels are the mean realized per capita transmission rate, $\hat{\beta }$, plotted against network size. Right-hand panels are the mean number of infections per edge between susceptible and infected nodes. Solid lines indicate a constant mean number of contacts for all population sizes. Dashed lines indicate a mean number of contacts that increase proportional to the square root of population size. Dotted lines indicate a mean number of contacts that increase linearly with the population size. Vertical bars give the standard deviation in observations from 30 simulated networks.

We find that the realized *per capita* transmission rate ($\hat{\beta }$) decreased with population size despite local transmission being modeled as a density-dependent process (Figures 3(a), 3(c), and 3(e)). Only when the mean number of contacts is assumed to increase linearly with population size is *per capita *rate ($\hat{\beta }$) constant across networks (Figures 3(a), 3(c), and 3(e)). Social group sizes that increase slower-than-linearly with community size yield intermediate results; $\hat{\beta }$ decays but slower than 1/*N*. In all cases, the local scale transmission remains constant across network sizes; that is, the number of new infections per susceptible-infected pair remains constant regardless of network size or configuration (Figures 3(b), 3(d), and 3(f)). Surprisingly, these scaling relationships are remarkably constant across the three classes of networks despite the difference in the variance in local contacts. For a given network size and mean number of contacts, the per capita transmission rate was lowest for the Poisson networks and greatest for the scale-free networks, in agreement with the standard observation that transmission is positively correlated with variance in the contact distribution. The critical finding from our analysis is that we need to distinguish between the *mode *of transmission within-populations (frequency versus density dependent) and the *scaling* of transmission between populations.

## 3. Discussion

Classically, the choice of model to describe disease dynamics was based on the transmission route of the pathogen, with little regard to empirical patterns. Even though this method may accurately describe local transmission between individual hosts, as evidenced by the wealth of empirical examples and the success of the resultant theory in disease management, it is not evident that these models will scale correctly to describe transmission across socially or spatially distinct populations (see examples in Table 1). In the above example, we have shown that the scaling pattern of pathogen transmission among distinct populations can be determined by the structure and scaling of the local host contact network and that the scaling pattern is independent of the mode of transmission. The scaling relationship depends explicitly on the heterogeneity in contacts and how the average connectivity changes with population size. In general, the scaling of transmission is likely to depend on the particular nature of host mixing and contact network structure—pathogen transmission biology may not play a large role. 

In all the models, the heterogeneity in the contact structure leads to a structured cascade of infection from highly connected individuals to less connected individuals [52], which results in a decline in the realized per capita transmission rate that is consistent with the predictions of the mean-field density-dependent model. (Note that the constant, or increasing, per capita transmission rate that is predicted by the mean-field, frequency-dependent model requires that new contacts be formed among individuals remaining in network model to overcome this frailty effect.) However, we only observed density-dependent scaling with population size in network models when the mean number of contacts is proportional to population size. This phenomenon is rarely observed in the literature (Table 1), possibly due to natural constraints on the number of contacts as populations grow, due to limited time or space. Presumably, for some very well-mixed systems such as phages in bacterial emulsions, density-dependent scaling with population size may be possible.

Despite locally density-dependent transmission, the network models predict $\hat{\beta }$ to scale in a pattern consistent with the frequency-dependent mean-field model when the mean number of social contacts is independent of population size or increases at a decelerating rate. We would expect this scaling pattern when social forces place constraints on the number of contacts independently of population size; for example, measles, for which school classroom sizes tend to be reasonably constrained [19], and phocine distemper virus in harbor seals, for which spatial constraints limit the number of seals on haulouts [18]. 

We further found that the scaling relationship was independent of the variance in the edge distribution of the contact network. Thus, the choice of the mean-field, density-dependent model to represent populations with homogeneous mixing [13], is not justified and would impose a scaling relationship that is only consistent with the extreme case where the mean number of contacts scale linearly with population size. As such, this observation corroborates the heuristic argument of Begon et al. [10] that the degree of local heterogeneity in the contact structure is “orthogonal to the distinction between density- and frequency-dependent contact rates and transmission.”

The network models we present here are necessarily simplistic in that they presume that epidemic dynamics are fast relative to ecological dynamics, so we can ignore births, nondisease mortality, and the formation of new connections following node removal (i.e., in the event that node removal is interpreted as mortality rather than lasting immunity). In principle, these should not impact our general observations providing that births and deaths are not biased with respect to the connectivity of nodes, an assumption implicit in the mean-field models. In practice, however, it is likely that births and deaths are biased with respect to network characteristics (e.g., [53]) or that connections are dynamic (connections accrue or change as nodes age [54]). Understanding these mechanisms that generate contact processes is an important ecological challenge and the efficient algorithms to incorporate these dynamics into model representations remain an important technical challenge. 

The density- and frequency-dependent, mean-field models make explicit assumptions about how the mean contact rate should scale with population density (or size if area is held constant as is the common assumption). As Begon et al. [10] point out, the direct application of these models across populations presents a challenge, as it requires the presumption that either area is comparable among populations of different size, or that the relationship between density and contact rate is consistent across populations. A variety of more flexible mean-field models have been proposed that allow for more intermediate, nonlinear relationships between density and contact rate [11]. However, while these intermediate relationships may provide better fit to observed dynamics, they are difficult to interpret in terms of explicit mechanisms, as there may be a range of candidate explanations for the nonlinear relationship between density and contacts [11]. 

Contact network models, while occasionally limiting in their complexity, present a useful tool for understanding the role of the social contact structure in generating observed dynamics. The focus on explicit characteristics of the contact structure provides a mechanistic explanation for the resulting dynamics compared with the phenomenological representations of contact rate assumed in the mean-field models. Here, we have shown the flexibility of these models to retain commonly observed within-population dynamics (i.e., the decline in realized transmission due to contact frailty) and a range of scaling patterns across population sizes as a function of the relationship between the contact distribution and the network size. We have presented only relatively simple examples where the mean and variance of the contact distribution scale with network size. However, it is reasonable to presume that a variety of additional, higher-order characteristics of contact networks (i.e., clustering, assortativity) may also scale with population size or density. The challenges of projecting even simple mean-field models across populations highlights the need for a greater understanding of how the characteristics of contact network correlate with more directly measureable metrics such as population size and density in order to make the lessons from contact network epidemiology predictive.

In conclusion, we must understand the local mixing dynamics of the host population rather than assume an implicit mixing structure defined by the pathogen in order to make predictions about epidemic dynamics across scales. Both experimental and theoretical work is needed to resolve the uncertainty about the scaling of transmission with population size and density. Experimentally, proper model systems will permit exploration of the effects of population size and density on mixing behavior and epidemic dynamics. Critically, this may depend strongly on behavioral responses to population size or density at the scale of individuals (e.g., [28]) that give rise to important deviations from the simple scaling models. Theoretically, method development and application will allow the study of contact network properties from field-sampled data on real populations. Network models in physics and epidemiology have provided great insights into the effect of heterogeneities and network topology on epidemic dynamics; however, connecting these insights to dynamics in real systems is challenging because of the need for fully censused populations to recreate contact networks and the assumption that network topology is static (though see [55]). The development of statistical methods to conduct inference on social network models [56] may prove useful to understand network assembly rules and for generating candidate networks for investigation of epidemic dynamics through simulation; in particular, how models of host movement and interaction among individuals give rise to scaling rules—be they network properties or mean-field approximations—for population mixing [57]. Overall, increasing the comprehension of host contact and mixing dynamics—through experimental and theoretical methods—will permit a more complete understanding of epidemic dynamics.

## Figures and Tables

**Table 1:** Empirical examples from the published literature of beta and *R*_0_ measured in host populations differing in size, indicating the empirical observations and the likely mean-field scaling model.

Host-pathogen system	Empirical observations	Model supported	Reference
Humans-measles Humans-pertussis Humans-diphtheria Humans-scarlet fever	Found *R*_0_ to be relatively invariant across population sizes.	Frequency dependent	[15]

Humans-smallpox	Transmission was inverse of population size	Frequency dependent	[58]

House finches-mycoplasma	Transmission was independent of flock sizes	Frequency dependent	[59]

Pigs-Aujeszky's disease virus (ADV)	*R*_0_ was invariant across different population sizes	Frequency dependent	[21]

Harbor seals-phocine distemper virus (PDV)	Density-dependent scaling did not explain differences in transmission between different-sized seal haul-out sites	Frequency dependent	[20]

Rana mucosa-chytridiomycosis	Transmission rate increases and saturates with density of infected individuals	Frequency dependent	[33]

Tasmanian devil—devil facial tumor disease	Maintenance of high prevalence following population decline	Frequency dependent	[34]

Brushtail possums-leptospira interogans	Density-dependent model fit experimental infection rates	Density dependent	[60]

Elk-brucellosis	Population density was associated with an increase in seroprevalence but could not differentiate among linear and nonlinear effects of host density.	Nonlinear density dependent	[61]

Rodents-cowpox	Both models fit to incidence time series; support for both equivocal.	Frequency and density dependent	[22]

Rodents-cowpox	Transmission term lies between density- and frequency-dependent and varies seasonally.	Model is intermediate	[11]

Indian meal moth-granulosis virus	A decline in transmission with increasing density of infectious cadavers	Neither	[26]

Possum-tuberculosis	Transmission did not fit frequency- or density-dependent models	Neither	[62]

Tiger salamander-*Abystomatigrinum* virus	Transmission was best modeled by a power or negative binomial function, that is, nonlinear density dependence.	Neither	[63]

Badgers-*Mycobacterium bovis *	Negative relationship between host abundance and infection prevalence	Neither	[64]
